# How Does Smartphone Use Impact Loneliness in the Post-COVID Landscape in Japan?

**DOI:** 10.3390/bs14040294

**Published:** 2024-04-03

**Authors:** Yu Kuramoto, Honoka Nabeshima, Mostafa Saidur Rahim Khan, Yoshihiko Kadoya

**Affiliations:** School of Economics, Hiroshima University, 1-2-1 Kagamiyama, Higashihiroshima 7398525, Hiroshima, Japan; b206431@hiroshima-u.ac.jp (Y.K.); b202314@hiroshima-u.ac.jp (H.N.); ykadoya@hiroshima-u.ac.jp (Y.K.)

**Keywords:** loneliness, smartphone use, COVID-19 pandemic

## Abstract

Smartphone use during the active phase of the COVID-19 pandemic emerged as a crucial means of facilitating communication when strict physical distancing was recommended. Previous studies conducted during the pandemic have suggested that smartphone use contributes to reduced loneliness. However, the influence of smartphone usage on the experience of loneliness in the aftermath of the active phase of the COVID-19 pandemic, also referred to as the post-COVID era, remains unclear, particularly because many physical communication restrictions were lifted during this period. To explore the association of smartphone use with the experience of loneliness in the post-COVID era, we analyzed the latest data from 2022 and 2023, when the COVID-19 pandemic gradually concluded. Our findings revealed that, in 2023, smartphone use increased the risk of loneliness among individuals aged 50–64 years. Conversely, among the younger generations, increased smartphone use was associated with decreased loneliness. The results of our study suggest that smartphones can serve as a significant tool for alleviating loneliness among the younger generations during the post-pandemic period.

## 1. Introduction

Recent research has shown that smartphone usage helped alleviate the risk of experiencing loneliness amid the COVID-19 pandemic [1,2]. These results contradict previous research before the pandemic, which demonstrated that greater smartphone usage increased the risk of loneliness [3,4]. The differences in the effects of smartphone use on loneliness before and during the pandemic motivated us to investigate whether smartphone use increased or decreased loneliness in the latest survey wave conducted in February 2023. During the pandemic, smartphones were used more because social distancing norms and national activity restrictions led to a predictable surge in digital technologies [5,6]. The frequency of smartphone use has increased to allow for communication without meeting in person, to gather information from the Internet, and to play games amid activity restrictions [6,7,8,9,10]. However, health and safety measures and economic activity have returned to some degree of normalcy due to the widespread use of vaccinations and government measures to combat the infection [11,12]. As opportunities to meet face-to-face have increased compared to during the pandemic, the role of smartphones as a means of maintaining social connections seems to have diminished. In this context of the change in smartphone usage and socioeconomic conditions compared to before or during the pandemic, assessing the recent association between smartphone use and loneliness is necessary. The recent wave of the Hiroshima University Household Behavioral and Financial Survey conducted in 2023 provides an exclusive opportunity to investigate how smartphone use impacts loneliness in Japan after the pandemic.

Numerous studies have examined the association between smartphone use and loneliness. Despite extensive research, conclusive longitudinal evidence on the contextual relationship between smartphones and loneliness remains lacking. Smartphone use has a two-way impact on loneliness [13], which can be explained by two hypotheses [14]. The “displacement hypothesis” proposes that loneliness may rise when online or digital media substitutes are used in place of face-to-face interaction within an offline setting [15,16,17]. On the other hand, the “stimulation hypothesis” assumes that people feel less lonely when using the Internet via mobile technology as it facilitates offline relationships and establishes new friendships [18,19,20].

Regarding the two-way association between loneliness and excessive use, extant studies offer stronger support for the displacement hypothesis than the stimulation hypothesis [18]. Most of these studies used cross-sectional data. Considering the intricate nature and divergent results of prior studies, longitudinal studies are required [1,21]. Thus, this study analyzed panel data from 2022 and 2023 to investigate the relationship between smartphones and loneliness after the COVID-19 pandemic.

In summary, there are notable gaps in research on how smartphone use is associated with loneliness. First, the influence of smartphone use on loneliness during the COVID-19 pandemic remains unclear. Second, there is scope for longitudinal research on the relationship between smartphone use and loneliness. To fill these gaps in the existing literature, we carried out a longitudinal empirical analysis using data from the 2020, 2022, and 2023 waves of the Hiroshima University Household Behavioral and Financial Panel Survey in Japan. Our objective was to examine whether the levels of loneliness changed when restrictive measures were lifted during the latter stages of the pandemic. Our hypothesis posits that the relationship between smartphone use and loneliness during the pandemic depends on the prevailing level of restrictive measures. Specifically, during periods of strict physical distancing, we anticipate that smartphone use will facilitate online communication as a means of maintaining social connections. However, during the latter phase of the pandemic, when such measures are relaxed, increased smartphone usage may increase loneliness because individuals continue to rely heavily on digital communication even when in-person interactions are feasible. Moreover, there is a fundamental human need for face-to-face communication, particularly after an extended period of limited social contact. Consequently, those who eschew such opportunities may experience increased feelings of loneliness.

This study makes three distinct contributions to the literature. First, to our knowledge, it is the inaugural examination of the correlation between smartphone use and loneliness in Japan, utilizing panel data gathered post-COVID-19 pandemic. Second, our study has implications for mental health care provision and treatment using digital technologies. Third, we revealed the impact of smartphone use, which varies by gender and age, via a subsample analysis.

## 2. Data and Methods

### 2.1. Data

Data used in this study has been collected from the 2020, 2022, and 2023 waves of the Household Behavioral and Financial Survey of Hiroshima University, conducted online by a renowned research company in Japan named Nikkei Research. The number of observations was calculated using random sampling to ensure representativeness. Participants older than 20 completed a questionnaire on their socio-demographic and psychological backgrounds and preferences. This survey was conducted over four years (2020, 2021, 2022, and 2023) before and during the COVID-19 pandemic. The initial wave was conducted between 20 and 25 February 2020, preceding the World Health Organization’s (WHO) declaration of COVID-19 as a global pandemic on 11 March 2020 [22]. The total number of participants was 17,463. The collection dates and sample sizes for the other three years were 19–26 February 2021 (6103 participants), 18–28 February 2022 (4281 participants), and 22 February–6 March 2023 (3410 participants). In this study, certain demographic variables were derived from the 2020 wave, such as male, age, children, living in rural areas, education, and financial literacy, while others were sourced from the 2022 and 2023 waves. After removing some missing variables, the final merged dataset for the three years consisted of 2952 observations.

### 2.2. Variables

The dependent variable of this study was the binary loneliness variable for the years 2022 and 2023. To assess this, we employed the UCLA method, consisting of three questions: “How frequently do you experience a sense of lacking companionship?”, “How often do you feel excluded?”, and “How often do you sense isolation from others?” Response choices comprised “hardly ever or never”, “some of the time”, and “often”. Participants were categorized as experiencing loneliness (loneliness = 1) if they responded “some of the time” or “often” to at least one question and as not lonely otherwise (loneliness = 0).

The primary independent variable was smartphone use, assessed in minutes per day with the question, “On average, how many hours do you use your smartphone per day?” Additionally, as control variables, we incorporated gender, age, presence of children, residence, years of education, and financial literacy, all of which served as indicators of rational financial and health behaviors [1,23,24], drawing from the 2020 dataset. For the 2022 and 2023 waves, we expanded the set of control variables to encompass marital status, living status, employment status, household financial status, subjective health status, depression, future anxiety, financial satisfaction, and a myopic view of the future. Our chosen variables align with those outlined by Nguyen et al. [1]. Detailed definitions for each variable are presented in Table 1 by Nguyen et al. [1].

### 2.3. Descriptive Statistics

The statistics provided in Table 2 reveal that 65.8% of the survey participants experienced loneliness between 2022 and 2023. On average, respondents spent 120 min per day on their smartphones. Nearly 59% of the participants were male, with an average age of 51 and an educational background spanning 15 years. Approximately 59.8% resided in rural areas, and 61.6% were engaged in full-time employment. The mean financial literacy score stood at 0.61 out of 1. Regarding household composition, 66.8% were married, 56.0% had children, and 20.0% lived independently. The majority of participants reported a total annual income of 6.41 million yen and a total asset value of 22.6 million yen. Lastly, respondents rated their subjective health status, future anxiety, financial satisfaction, depression, and myopic views of the future at 3.27, 3.79, 2.81, 2.88, and 2.72 out of 5, respectively. 

The whole sample was stratified into sex- and age-based subsamples, and the *t*-test and ANOVA were performed, respectively, as presented in Table 3 and Table 4. These tests did not demonstrate statistically significant differences between the sexes or across age groups. 

### 2.4. Methods

The objective of this study is to conduct a longitudinal analysis that examines the relationship between smartphone use and loneliness. Our hypothesis suggests that the impact of smartphone use on loneliness during the pandemic is contingent on the level of restrictive measures in place during various phases. When physical communication is heavily restricted, we anticipate that smartphone usage will serve as a means to maintain social connections via online communication. However, increased smartphone usage during the latter stages of the pandemic, when restrictive measures are relaxed, may lead to increased feelings of loneliness as individuals continue to rely heavily on digital communication even when face-to-face interaction is feasible. To test this hypothesis, we employed a binary variable representing respondents’ feelings of loneliness during different years of the pandemic as the dependent variable, with the usage time of smartphones serving as the independent variable. Additionally, we controlled for various demographic and socioeconomic factors that could potentially influence loneliness. The following equation was utilized to investigate the longitudinal association between smartphone use and loneliness:

(1) $$Y_{it}=f\left({{SU}_{it},\;X}_{it},\;{u_{i},\varepsilon }_{it}\right)$$

where $Y_{it}$ indicates whether the ith respondent is lonely in t year (t=2022 or t=2023). SU represents how long a respondent spent using a smartphone. X is a vector of individual demographic, socioeconomic, psychological, and health characteristics; u represents the individual effect, and ε is the error term. As our dependent variable was binary, and this study used panel data, we performed random- and fixed-effect logit model analyses. 

We conducted a Hausman test to assess whether the random- or fixed-effect models were more appropriate. The results of the Hausman test (available upon request) indicate that the random effects model should be applied in this study. However, in addition to the random-effect model, we report the results of the fixed-effect model, which accounts for correlations between individual heterogeneous factors and independent variables. This is because our dependent variable (loneliness) is subjective, and how smartphone use affects loneliness depends on individual factors such as personality.

As our regression results were vulnerable to a multicollinearity problem, we also tested for correlation and multicollinearity. Our results indicated a mild correlation between the relative changes in the two variables (less than 0.70). Additionally, the variance inflation factor (VIF) values for all independent variables were below three, suggesting the absence of multicollinearity in any model (results available upon request). 

The full specification for Equation (1) is as follows:

(2) $$\begin{array}{ll} {Loneliness}_{it}= & \beta_{0}+\beta_{1}{Smartphone\;use}_{it}+\beta_{2}{Male}_{it}+\beta_{3}{Age}_{it}+\beta_{4}{Age\;squared}_{it}+\beta_{5}{Spouse}_{it}\\ & +\beta_{6}{Children}_{it}+\beta_{7}{Living\;alone}_{it}+\beta_{8}{Living\;in\;rural\;areas}_{it}+\beta_{9}{Education}_{it}\\ & +\beta_{10}{Full-time\;employment}_{it}+\beta_{11}{Log\;of\;household\;income}_{it}\\ & +\beta_{12}{Log\;of\;household\;assets}_{it}+\beta_{13}{Financial\;literacy}_{it}+\beta_{14}{Subject\;health\;status}_{it}\\ & +\beta_{15}{Future\;anxiety}_{it}+\beta_{16}{Financial\;statisfaction}_{it}+\beta_{17}{Depression}_{it}\\ & +\beta_{18}{Myopic\;view\;of\;the\;future}_{it}+u_{i}+\varepsilon_{it} \end{array}$$

## 3. Results

The results of the random- and fixed-effect models for the full sample are presented in Table 5 and Table 6. Although our preferred model, as indicated by the Hausman test, is the random-effect model, we also reported the fixed-effect model to ensure the robustness of the results, as it allows for individual heterogeneity. In Models 1–3 of the random-effect model, which did not control depression and a myopic view of the future, we observe a positive association between smartphone use and loneliness. However, this association loses significance when psychological factors such as depression and a myopic view of the future are controlled. Furthermore, our findings indicate that having a spouse, subjective health status, and financial satisfaction are negatively associated with loneliness, whereas living alone, anxiety, depression, and a myopic view of the future are positively associated. Additionally, our study suggests that such factors as gender, age, having children, residing in rural areas, education level, employment status, household income and assets, and financial literacy do not exhibit a significant association with loneliness.

Similarly, the fixed-effect model demonstrates that the association between smartphone use and loneliness becomes insignificant after controlling for individual characteristics. Among the control variables, living alone and depression exhibit positive associations with loneliness, while subjective health status and financial satisfaction exhibit negative associations. The remaining control variables do not show a significant association with loneliness.

Thus, both the random-effect and fixed-effect models yield consistent results regarding the association between smartphone use and loneliness.

Subsample analyses were conducted based on sex and age to better understand the relationship between loneliness and socioeconomic factors, given previous studies indicating their significant influence on loneliness [1]. The results of the subsample analyses are presented in Table 7, Table 8, Table 9 and Table 10. Specifically, Table 7 and Table 9 display the results of gender-based subsample analyses for the random-effect and fixed-effect models, respectively, while Table 8 and Table 10 present age-based subsample analyses for the random-effect and fixed-effect models, respectively.

In the gender-based subsample analysis, the results of the random-effect model indicate that the association between smartphone use and loneliness is not significant for either males or females. In particular, differences in influential factors for male and female loneliness are observed among the control variables. For males, living alone, future anxiety, and depression exhibit positive associations with loneliness while having a spouse, subjective health status, and financial satisfaction display negative associations. Conversely, for females, financial literacy, future anxiety, and depression are positively associated with loneliness, while education, subjective health status, and financial satisfaction show negative associations. Similarly, the results of the fixed-effect model yield similar findings, with no significant association between smartphone use and loneliness for either gender, and consistency observed in the significance of control variables compared to the random-effect models.

Regarding the age-based subsample analysis, the results of the random-effect model show a negative association between smartphone use and loneliness for the younger subsample aged 34 and below, while a positive association is observed for the relatively older subsample aged between 50 and 64. Across all age groups, future anxiety and depression exhibit positive associations with loneliness, whereas other factors such as living alone, education, financial literacy, subjective health status, and financial satisfaction show inconsistent associations with loneliness. However, the fixed-effect model does not confirm the association between smartphone use and loneliness in any age group. Among the control variables, living alone, employment, subjective health status, and a myopic view of the future display inconsistent associations, while depression consistently relates to loneliness across all age groups.

## 4. Discussion 

The influence of smartphones on feelings of loneliness is still an inconclusive issue. Smartphones in relation to loneliness became important during the pandemic when social isolation was strictly recommended. According to a recent study, the use of smartphones was associated with a reduction in feelings of loneliness amid the COVID-19 pandemic [1]. However, the positive influence of smartphone use on loneliness was not observed before the pandemic [3]. This inconsistency led us to conduct this longitudinal study to understand whether the influence of smartphone use has changed further amidst the withdrawal of social and physical restrictions. Using data from 2022 and 2023, after the end of the COVID-19 pandemic, our analysis revealed that increased smartphone usage increased loneliness among individuals aged 50–64, while it reduced loneliness for the younger generation of under 34. These findings partially support our hypothesis, suggesting that increased smartphone use tends to exacerbate loneliness when opportunities for physical communication are available. In this regard, our results align with the findings of Lapierre et al. [3], who provided longitudinal evidence of increased loneliness due to excessive smartphone use before the pandemic. However, the findings also partially contradict our hypothesis regarding the continued use of smartphones by the younger generation even after the withdrawal of restrictive measures but experiencing reduced loneliness. This finding supports the earlier study that indicated a negative relationship between loneliness and smartphone use during the COVID-19 pandemic [1]. 

It is important to explore why the withdrawal of restrictive measures had differing impacts on younger and older generations. Our results suggest that older generations are embracing the new era of unrestricted physical communication as a means of fulfilling their social needs, thus reducing their loneliness. Conversely, the younger generation appears to be less inclined to utilize unrestricted physical communication for socialization, instead continuing to seek support from digital platforms for social interaction and mental well-being. This suggests that the younger generation is not fully taking advantage of the opportunity for unrestricted physical communication for socialization compared to their older counterparts after the pandemic [25,26]. This explanation aligns with the findings of Bjørknes et al. [27], who observed that young people perceived this period as an irreversible loss of youth and felt demotivated to reintegrate into normal society even after the pandemic.

Among the control variables, having a spouse, reporting sound subjective health status, and experiencing higher levels of financial satisfaction are likely to decrease loneliness, whereas living alone, experiencing future anxiety, depression, and having a myopic view of the future are likely to increase loneliness. These results align with earlier studies, which provide evidence that increased interaction with family and friends, along with experiencing less financial and non-financial anxiety, contribute to reduced loneliness, while higher levels of mental health conditions such as depression and health concerns can increase loneliness [1]. However, it is important to note that loneliness is a socio-psychological issue wherein individuals may feel lonely even when surrounded by family and friends. The feeling of being left out can arise from various factors, including family and social interactions, as well as health and financial status.

There are several limitations to this study. First, the data did not provide details on the frequency or purpose of smartphone usage. Second, the government of Japan officially withdrew the pandemic status of COVID-19 in May 2023. Therefore, it may not be possible to say that the pandemic ended completely in February 2023, when we collected the data. Despite these limitations, our findings have significant implications for policymakers. In particular, younger generations, who have lagged in reintegrating into society after the pandemic, should have been offered more opportunities to improve their loneliness and mental health.

## 5. Conclusions

Our study reveals the evolving dynamics of smartphone usage and its influence on loneliness in the post-COVID era. While smartphones were recognized as vital communication tools during the active phase of the pandemic, our investigation of their use after the pandemic revealed nuanced effects on loneliness. We used a panel survey and utilized mean comparison tests and panel regression analyses to provide longitudinal evidence on the association between smartphone use and loneliness, particularly in the post-COVID era.

The univariate analysis reveals that during the post-COVID period, approximately 66% of respondents reported feelings of loneliness. Concurrently, the respondents reported an average smartphone usage time of around 120 min or 2 h per day. The pronounced levels of loneliness and smartphone usage require further investigation of their true association. Unlike previous studies conducted during the pandemic, our findings from 2022 to 2023 indicate a change in the relationship between smartphone use and loneliness. Notably, by 2023, we observed an increased risk of loneliness associated with smartphone use among individuals aged 50–64. This suggests a potential vulnerability in this age group, possibly influenced by the changing communication patterns or other factors after the pandemic. Conversely, younger generations demonstrated a contrasting pattern, with increased smartphone use linked to decreased loneliness. This intriguing disparity highlights the generational divide in the perceived impact of smartphones on social connections and mental well-being in the post-COVID period. Among the control variables, future anxiety, depression, and a myopic view of the future were identified as factors that increased loneliness, while having a spouse, reporting sound health status, and experiencing financial satisfaction consistently reduced loneliness.

Our results underscore the importance of considering age-specific nuances when examining the role of smartphones in mitigating loneliness. As the COVID-19 pandemic has gradually ended, smartphones have emerged as a significant tool to alleviate loneliness among younger generations. However, the complexities revealed in our study emphasize the need for ongoing research to understand and adopt strategies that effectively harness the potential of smartphones to promote mental well-being across different age groups within an evolving post-pandemic landscape.

## Figures and Tables

**Table 1:** Variable definitions.

Variables	Definition
Dependent variable	
Loneliness	Binary variable where 1 indicates that participants have feelings of loneliness some of the time or often and 0 otherwise
Independent variables	
Smartphone use	Continuous variable indicating respondent’s use of smartphone in number of minutes/day
Male *	Binary variable where 0 = female, and 1 = male
Age *	Continuous variable indicating age of respondents
Age squared *	Continuous variable indicating age squared of respondents
Spouse	Binary variable where 1 = respondents have a spouse or partner; 0 = otherwise
Children *	Binary variable where 1 = respondents have at least one child; 0 = having no child
Living alone	Binary variable where 1 = respondents live alone; 0 = otherwise
Living in rural areas *	Binary variable where 1 = respondents live in rural areas (not in Tokyo special wards or government-designated city areas); 0 = otherwise
Education *	Discrete variable indicating years of education of respondents
Full-time employment	Binary variable where 1 = respondents have a full-time job; 0 = otherwise
Household income	Continuous variable indicating annual earned income before taxes and with bonuses of the entire household (unit: JPY)
Log of household income	Log (household income)
Household asset	Continuous variable indicating balance of financial assets (savings, stocks, bonds, insurance, etc.) of the entire household (unit: JPY)
Log of household asset	Log (household asset)
Financial literacy *	Continuous variable indicating average scores of respondents obtained from answering the three financial literacy questions
Subjective health status	Ordinal variable: “I am now healthy and was generally healthy in the past year”. Variable 5 = it is particularly true; 4 = it is rather true; 3 = neither true nor untrue; 2 = it is not so true; 1 = it is not true at all.
Future anxiety	Ordinal variable: “I have anxieties about life after 65 years of age” and “I have anxieties about life in the future” for individuals under 65 and for those who were aged 65 or older, respectively. Variable 5 = it is particularly true; 4 = it is rather true; 3 = neither true nor untrue; 2 = it is not so true; 1 = it is not true at all.
Financial satisfaction	Ordinal variable: “I am happy with my financial status”. Variable 5 = completely agree; 4 = agree; 3 = neither agree nor disagree; 2 = disagree; 1 = disagree completely.
Depression	Ordinal variable: “I often feel depressed or felt depressed in the past year”. Variable 5 = it is particularly true; 4 = it is rather true; 3 = neither true nor untrue; 2 = it is not so true for you, 1 = it does not hold true at all for you.
Myopic view of the future	Ordinal variable: “Since the future is uncertain, it is a waste to think about it”. Variable 5 = completely agree; 4 = agree; 3 = neither agree nor disagree; 2 = disagree; 1 = disagree completely.

* Indicates data from the 2020 wave.

**Table 2:** Descriptive statistics.

Variables	Mean	Std. Dev.	Min	Max
Dependent variable				
Loneliness	0.6579	0.4745	0	1
Independent variable				
Smartphone use	120.3930	130.6524	0	1440
Male	0.5881	0.4923	0	1
Age	51.1579	14.1853	22	91
Age squared	2818.2800	1490.2550	484	8281
Spouse	0.6680	0.4710	0	1
Children	0.5596	0.4965	0	1
Living alone	0.2005	0.4005	0	1
Living in rural areas	0.5982	0.4903	0	1
Education	14.7622	2.0869	9	21
Full-time employment	0.6162	0.4864	0	1
Household income	6,412,602	4,304,626	500,000	21,000,000
Log of household income	15.4157	0.7988	13.12	16.86
Household asset	22,600,000	30,800,000	1,250,000	125,000,000
Log of household asset	16.0301	1.4350	14.04	18.64
Financial literacy	0.6086	0.3836	0	1
Subjective health status	3.2700	1.0955	1	5
Future anxiety	3.7886	1.1285	1	5
Financial satisfaction	2.8194	1.0966	1	5
Depression	2.8764	1.2422	1	5
Myopic view of the future	2.7161	0.9862	1	5
Observation	2952			

**Table 3:** Loneliness distribution across gender.

Loneliness	Gender		Total
	Male	Female	
No	590	420	1010
	33.99%	34.54%	34.21%
Yes	1146	796	1942
	66.01%	65.46%	65.79%
Total	1736	1216	2952
	100%	100%	100%
Mean Different	t = −0.3118		

**Table 4:** Loneliness distribution across gender age groups.

Loneliness	Age				
	≤34	35–49	50–64	≥65	Total
No	174	323	315	198	1010
	36.71%	32.20%	33.58%	36.87%	34.21%
Yes	300	680	623	339	1942
	63.29%	67.80%	66.42%	63.13%	65.79%
Total	474	1003	938	537	2952
	100%	100%	100%	100%	100%
F-statistics	F = 1.66				

**Table 5:** A random effect logit regression results of loneliness.

Variables	Dependent Variable: Loneliness			
	Model_1	Model_2	Model_3	Model_4
Smartphone use	0.0008 **	0.0007 **	0.0006 *	0.0003
	(0.0003)	(0.0003)	(0.0003)	(0.0004)
Male	0.0528	0.0396	0.0544	0.0545
	(0.0922)	(0.0951)	(0.0992)	(0.1051)
Age	0.0288	0.0295	0.0318	0.0292
	(0.0188)	(0.0191)	(0.0200)	(0.0212)
Age squared	−0.0003 *	−0.0003 *	−0.0003 *	−0.0003
	(0.0002)	(0.0002)	(0.0002)	(0.0002)
Spouse	−0.6464 ***	−0.5594 ***	−0.4079 ***	−0.3464 **
	(0.1258)	(0.1291)	(0.1351)	(0.1423)
Children	−0.0610	−0.0779	−0.0609	−0.0772
	(0.0907)	(0.0914)	(0.0956)	(0.1014)
Living alone	0.2084	0.1476	0.2860 *	0.3723 **
	(0.1514)	(0.1541)	(0.1610)	(0.1699)
Living in rural areas	−0.0230	−0.0064	0.0156	−0.0143
	(0.0852)	(0.0859)	(0.0898)	(0.0952)
Education	−0.0225	−0.0289	−0.0221	−0.0319
	(0.0203)	(0.0210)	(0.0220)	(0.0233)
Full-time employment		0.0626	0.0148	−0.0144
		(0.0932)	(0.0975)	(0.1024)
Log of household income		−0.1305 *	−0.0460	−0.0318
		(0.0691)	(0.0722)	(0.0760)
Log of household asset		−0.0998 ***	0.0354	0.0465
		(0.0327)	(0.0359)	(0.0381)
Financial literacy		0.0717	0.0682	0.0817
		(0.1193)	(0.1248)	(0.1322)
Subjective health status			−0.2446 ***	−0.1469 ***
			(0.0422)	(0.0449)
Future anxiety			0.3141 ***	0.1788 ***
			(0.0441)	(0.0471)
Financial satisfaction			−0.2011 ***	−0.1472 ***
			(0.0492)	(0.0524)
Depression				0.5010 ***
				(0.0465)
Myopic view of the future				0.0845 *
				(0.0469)
lnsig2u	−1.7761**	−1.7208 **	−1.4337 **	−0.9801 **
	(0.8044)	(0.7764)	(0.6379)	(0.4670)
Constant	0.7097	4.2998 ***	0.8036	−0.9427
	(0.5714)	(1.0969)	(1.1897)	(1.2817)
Observations	2952	2952	2952	2952
Number of record_id	1476	1476	1476	1476
Log-likelihood	−1852	−1840	−1756	−1681
Chi2 statistics	77.40	94.96	201.6	259.3
*p*-value	0	0	0	0

Standard errors are shown in parentheses. *** *p* < 0.01, ** *p* < 0.05, and * *p* < 0.1 indicate the level of significance.

**Table 6:** A fixed-effect logit regression results of loneliness.

Variables	Dependent Variable: Loneliness			
	Model_1	Model_2	Model_3	Model_4
Smartphone use	0.0011 **	0.0010 **	0.0009 *	0.0006
	(0.0005)	(0.0005)	(0.0005)	(0.0005)
Male	-	-	-	-
Age	0.1631	0.1381	0.2738	0.1202
	(0.3011)	(0.3069)	(0.3311)	(0.3575)
Age squared	−0.0009	−0.0007	−0.0021	−0.0000
	(0.0029)	(0.0029)	(0.0031)	(0.0034)
Spouse	−0.7021 ***	−0.6130 ***	−0.3724 *	−0.2302
	(0.1773)	(0.1820)	(0.1957)	(0.2130)
Children	-	-	-	-
Living alone	0.2678	0.2241	0.4223 *	0.5999 **
	(0.2070)	(0.2132)	(0.2282)	(0.2519)
Living in rural areas	-	-	-	-
Education	-	-	-	-
Full-time employment		−0.0161	−0.0846	−0.1295
		(0.1370)	(0.1477)	(0.1592)
Log of household income		−0.1401	−0.0120	0.0402
		(0.0979)	(0.1075)	(0.1164)
Log of household asset		−0.1347 ***	−0.0244	−0.0083
		(0.0451)	(0.0508)	(0.0551)
Financial literacy		-	-	-
Subjective health status			−0.2921 ***	−0.1578 **
			(0.0616)	(0.0685)
Future anxiety			0.2848 ***	0.0923
			(0.0635)	(0.0709)
Financial satisfaction			−0.1941 ***	−0.1600 **
			(0.0721)	(0.0790)
Depression				0.5882 ***
				(0.0692)
Myopic view of the future				0.0944
				(0.0689)
Observations	1292	1292	1292	1292
Number of record_id	646	646	646	646
Log-likelihood	−419.1	−410.3	−370	−324
Chi2 statistics	57.27	74.94	155.6	247.5
*p*-value	0	0	0	0

Standard errors are shown in parentheses. *** *p* < 0.01, ** *p* < 0.05, and * *p* < 0.1 indicate the level of significance.

**Table 7:** A random effect logit regression results of loneliness (subsample analysis by gender).

Variables	Dependent Variable: Loneliness	
	Male	Female
Smartphone use	0.0004	0.0001
	(0.0005)	(0.0005)
Age	0.0459	0.0437
	(0.0338)	(0.0313)
Age squared	−0.0004	−0.0005 *
	(0.0003)	(0.0003)
Spouse	−0.3619 *	−0.3204
	(0.1999)	(0.2054)
Children	−0.0568	−0.1294
	(0.1493)	(0.1391)
Living alone	0.4881 **	0.2615
	(0.2391)	(0.2470)
Living in rural areas	−0.1236	0.1226
	(0.1379)	(0.1331)
Education	−0.0101	−0.0793 **
	(0.0322)	(0.0357)
Full-time employment	−0.0622	0.0305
	(0.1439)	(0.1476)
Log of household income	0.0686	−0.1510
	(0.1077)	(0.1090)
Log of household asset	0.0505	0.0445
	(0.0535)	(0.0550)
Financial literacy	−0.1166	0.3369 *
	(0.1980)	(0.1799)
Subjective health status	−0.1640 ***	−0.1297 **
	(0.0630)	(0.0646)
Future anxiety	0.1934 ***	0.1556 **
	(0.0658)	(0.0681)
Financial satisfaction	−0.1368 *	−0.1738 **
	(0.0742)	(0.0753)
Depression	0.6096 ***	0.3774 ***
	(0.0677)	(0.0625)
Myopic view of the future	0.1076	0.0500
	(0.0667)	(0.0656)
lnsig2u	−0.2461	−10.1649
	(0.3814)	(12.7719)
Constant	−3.5458 *	1.8105
	(1.8553)	(1.8614)
Observations	1736	1216
Number of record_id	868	608
Log-likelihood	−967.5	−702.2
Chi2 statistics	159.5	133.3
*p*-value	0	0

Standard errors are shown in parentheses. *** *p* < 0.01, ** *p* < 0.05, and * *p* < 0.1 indicate the level of significance.

**Table 8:** A random effect logit regression results of loneliness (subsample analysis by age group).

Variables	Dependent Variable: Loneliness			
	≤34	35–49	50–64	≥65
Smartphone use	−0.0013 *	−0.0001	0.0016 **	0.0010
	(0.0007)	(0.0006)	(0.0008)	(0.0008)
Male	0.0855	−0.1779	0.3043	0.3262
	(0.2435)	(0.1577)	(0.2129)	(0.2977)
Spouse	−0.5709 *	−0.1981	−0.2741	−0.3385
	(0.3348)	(0.2273)	(0.2963)	(0.3360)
Children	−0.1646	−0.1355	−0.0330	−0.3763
	(0.2358)	(0.1519)	(0.1954)	(0.3346)
Living alone	−0.0999	0.5981 **	0.9271 ***	−0.2593
	(0.3999)	(0.2794)	(0.3595)	(0.3941)
Living in rural areas	−0.2147	−0.0193	0.2205	−0.1990
	(0.2150)	(0.1562)	(0.1967)	(0.2222)
Education	0.0268	−0.0499	−0.1310 ***	0.0816
	(0.0564)	(0.0370)	(0.0503)	(0.0543)
Full-time employment	0.1251	−0.0555	0.0049	0.0461
	(0.2393)	(0.1677)	(0.2024)	(0.2506)
Log of household income	−0.1209	−0.1206	0.0485	0.0010
	(0.1636)	(0.1253)	(0.1538)	(0.1935)
Log of household asset	0.1296	−0.0035	0.1131	−0.0096
	(0.0893)	(0.0609)	(0.0772)	(0.0954)
Financial literacy	−0.5444 *	0.5247 **	0.0670	−0.1344
	(0.3016)	(0.2128)	(0.2642)	(0.3413)
Subjective health status	−0.1389	0.0058	−0.2777 ***	−0.2866 ***
	(0.1055)	(0.0746)	(0.0942)	(0.1014)
Future anxiety	0.2874 **	0.1762 **	0.1584 *	0.1833 *
	(0.1157)	(0.0782)	(0.0938)	(0.1084)
Financial satisfaction	−0.1170	−0.1203	−0.2923 ***	−0.0449
	(0.1230)	(0.0863)	(0.1053)	(0.1269)
Depression	0.3651 ***	0.5168 ***	0.5817 ***	0.5063 ***
	(0.1019)	(0.0790)	(0.0969)	(0.1035)
Myopic view of the future	0.0623	0.1144	0.0627	0.0597
	(0.1114)	(0.0766)	(0.0945)	(0.1108)
lnsig2u	−12.3465	−3.6539	0.0505	−1.1094
	(44.3521)	(10.0552)	(0.4552)	(1.2196)
Constant	−0.6573	1.3213	−0.9128	−1.1860
	(2.6500)	(1.9858)	(2.3298)	(2.8553)
Observations	474	1003	938	537
Number of record_id	245	538	508	279
Log-likelihood	−277.8	−549.2	−516.3	−305.2
Chi2 statistics	55.93	93.25	80.86	54.20
*p*-value	2.50 × 10^−6^	0	1.16 × 10^−10^	4.81 × 10^−6^

Standard errors are shown in parentheses. *** *p* < 0.01, ** *p* < 0.05, and * *p* < 0.1 indicate the level of significance.

**Table 9:** A fixed-effect logit regression results of loneliness (subsample analysis by gender).

Variables	Dependent Variable: Loneliness	
	Male	Female
Smartphone use	0.0007	0.0007
	(0.0008)	(0.0007)
Age	0.4799	0.1358
	(0.5824)	(0.5126)
Age squared	−0.0018	−0.0022
	(0.0051)	(0.0055)
Spouse	−0.1756	−0.2900
	(0.3176)	(0.2997)
Children	-	-
Living alone	0.9953 ***	0.2601
	(0.3835)	(0.3450)
Living in rural areas	-	-
Education	-	-
Full-time employment	−0.2855	0.0521
	(0.2294)	(0.2314)
Log of household income	0.1799	−0.0843
	(0.1724)	(0.1674)
Log of household asset	0.0070	−0.0299
	(0.0818)	(0.0783)
Financial literacy	-	-
Subjective health status	−0.0603	−0.2700 ***
	(0.1009)	(0.0983)
Future anxiety	0.0925	0.0927
	(0.0981)	(0.1100)
Financial satisfaction	−0.2825 **	−0.0920
	(0.1183)	(0.1137)
Depression	0.7507 ***	0.4223 ***
	(0.1023)	(0.0992)
Myopic view of the future	0.0476	0.1324
	(0.0990)	(0.1001)
lnsig2u	-	-
Constant	-	-
Observations	716	576
Number of record_id	358	288
Log-likelihood	−163.2	−153.2
Chi2 statistics	170	92.82
*p*-value	0	0

Standard errors are shown in parentheses. *** *p* < 0.01 and ** *p* < 0.05 indicate the level of significance.

**Table 10:** A fixed-effect logit regression results of loneliness (subsample analysis by age group).

Variables	Dependent Variable: Loneliness			
	≤34	35–49	50–64	≥65
Smartphone use	0.0001	−0.0002	0.0020	0.0013
	(0.0011)	(0.0011)	(0.0013)	(0.0016)
Male	-	-	-	-
Spouse	−0.4069	−0.0056	−0.1573	−0.4607
	(0.4514)	(0.4271)	(0.5023)	(0.4975)
Children	-	-	-	-
Living alone	−0.2097	1.1115 **	1.0695 *	−0.4325
	(0.5674)	(0.4896)	(0.6007)	(0.7030)
Living in rural areas	-	-	-	-
Education	-	-	-	-
Full-time employment	−0.1845	−0.5454 *	0.5144	−0.0588
	(0.3657)	(0.3078)	(0.3245)	(0.4770)
Log of household income	0.1266	0.2200	−0.0234	−0.3364
	(0.2440)	(0.2059)	(0.2520)	(0.3518)
Log of household asset	0.0485	−0.1152	0.0831	−0.0514
	(0.1262)	(0.1018)	(0.1143)	(0.1527)
Financial literacy	-	-	-	-
Subjective health status	−0.1199	−0.1317	−0.2378	−0.3547 **
	(0.1620)	(0.1372)	(0.1468)	(0.1626)
Future anxiety	0.1734	−0.0232	0.1880	0.1661
	(0.1903)	(0.1381)	(0.1502)	(0.1547)
Financial satisfaction	−0.2528	−0.1304	−0.2287	0.0322
	(0.2024)	(0.1458)	(0.1706)	(0.1918)
Depression	0.4735 ***	0.7095 ***	0.6479 ***	0.5816 ***
	(0.1719)	(0.1299)	(0.1452)	(0.1644)
Myopic view of the future	−0.0695	0.3332 **	0.1006	−0.1989
	(0.1807)	(0.1328)	(0.1425)	(0.1789)
lnsig2u	-	-	-	-
Constant	-	-	-	-
Observations	224	416	338	232
Number of record_id	112	208	169	116
Log-likelihood	−60.52	−95.22	−79.21	−56.31
Chi2 statistics	34.23	97.91	75.87	48.19
*p*-value	0.000331	0	0	1.32 × 10^−6^

Standard errors are shown in parentheses. *** *p* < 0.01, ** *p* < 0.05, and * *p* < 0.1 indicate the level of significance.

## Data Availability

Data are available upon request.
